# Case Report: Organizing pneumonia associated with AQP4-antibody neuromyelitis optica spectrum disorder in children

**DOI:** 10.3389/fped.2025.1729466

**Published:** 2026-01-13

**Authors:** Lijun Zhang, Yixuan Liu, Yakun Wang, Liyuan Tian

**Affiliations:** Department of Respirology, Hebei children’s Hospital, Hebei Clinical Medicine Research Center for Children’s Health and Diseases, Shijiazhuang, China

**Keywords:** antibody, AQP4, children, NMOSD, organizing pneumonia

## Abstract

**Background:**

Aquaporin-4-antibody Neuromyelitis Optica Spectrum Disorder (AQP4-Ab NMOSD) is an immune-mediated inflammatory disorder of the central nervous system, characterized by predominant involvement of the optic nerves and spinal cord. Pulmonary involvement, particularly in the form of organizing pneumonia, remains an uncommon manifestation of AQP4-Ab NMOSD.

**Case summary:**

We present the case of an 8-year-old girl with AQP4-Ab NMOSD who developed organizing pneumonia concurrent with the onset of her neurological disease. The initial lung injury resolved following immunotherapy, while the subsequent injury improved spontaneously. Anti-AQP4-IgG Antibody was detected positive in the bronchoalveolar lavage fluid.

**Conclusion:**

When lung injury occurs in patients with AQP4-Ab NMOSD, detection of anti-AQP4-IgG antibodies in bronchoalveolar lavage fluid may serve as an adjunctive diagnostic tool for the organizing pneumonia associated with AQP4-Ab NMOSD.

## Introduction

Aquaporin-4-antibody Neuromyelitis Optica Spectrum Disorder(AQP4-Ab NMOSD) is an immune-mediated inflammatory disorder of the central nervous system, characterized primarily by attacks affecting the optic nerve, spinal cord and area postrema (1). The discovery of Neuromyelitis optica spectrum disorder (NMOSD) can be traced back to 1894, when Dr Eugène Devic and his doctoral student Fernand Gault first delineated the condition (2). The global prevalence of NMOSD ranges from 0.5 to 10 cases per 100,000 person-years (3), with higher susceptibility observed in non-Caucasian populations (4). In 2020, China reported NMOSD hospitalization data derived from its inpatient registry system (2016–2018), revealing an overall incidence rate of approximately 0.278 per 100,000 person-years. The incidence rates were 0.348 per 100,000 person-years in adults and 0.075 per 100,000 person-years in children (5).

The pathogenesis of NMOSD is primarily mediated by pathogenic aquaporin-4(AQP4) immunoglobulin G autoantibodies, which target astrocytes in the central nervous system, leading to severe axonal injury and demyelination (6). Interestingly, AQP4 expression has been identified in lung tissue (7). Although a link between organizing pneumonia and NMOSD has been recognized, documented cases are exceedingly rare (8), and there is a lack of adjunctive diagnostic tool. Herein, we report an 8-year-old girl with AQP4-Ab NMOSD who developed organizing pneumonia, suggesting lung involvement, concurrent with the onset of her neurological disease.

## Case presentation

**Figure 1:**
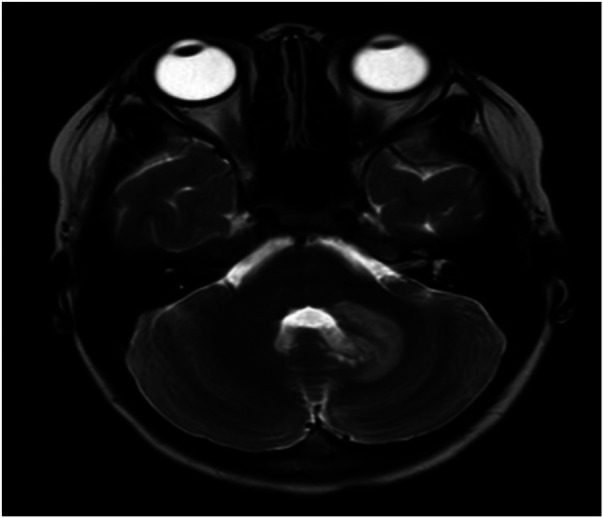
MRI T2-weighted imaging revealed an area of hyperintensity in the left cerebellar hemisphere.

**Figure 2:**
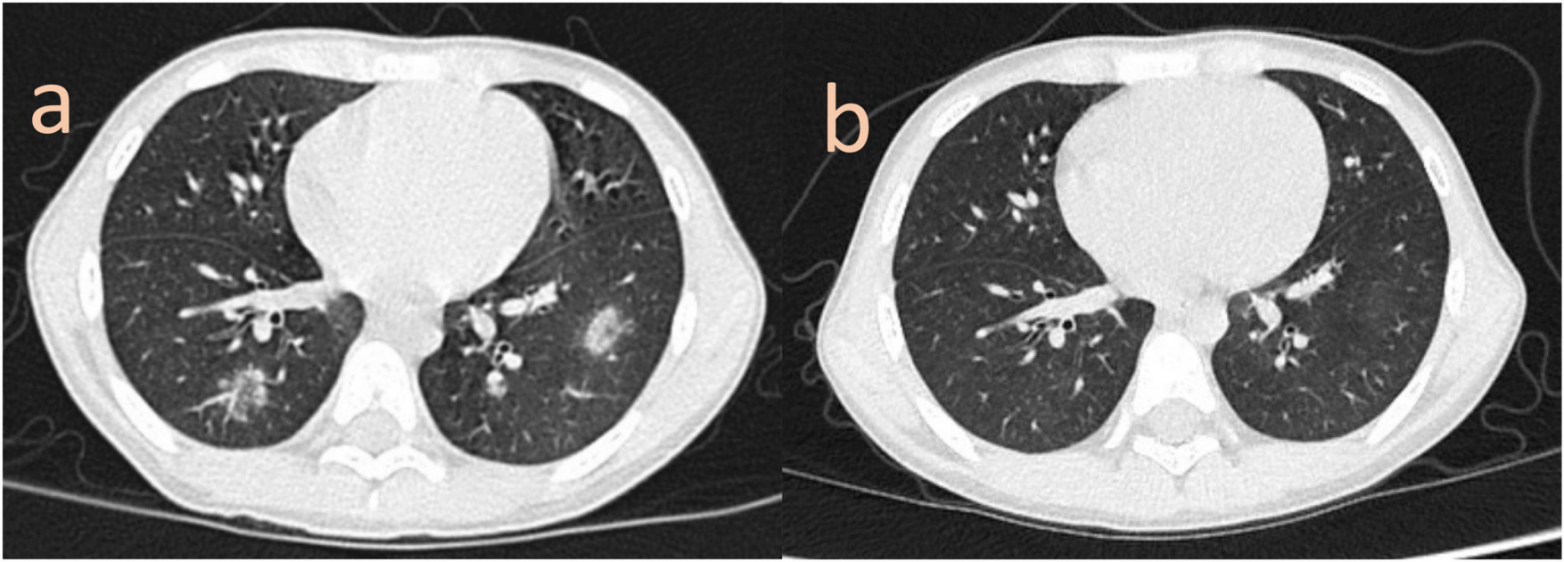
**(a)** peripheral ground-glass opacities were observed in the lower lobes prior to immunosuppressive therapy, some of which were characterized by the reverse halo sign. **(b)** One-month follow-up revealed near-complete resolution of the prior opacities.

An 8-year-old girl presented with a one-month history of dizziness, vomiting, hand tremors, and an unsteady gait in October 2023. The patient had a history of head trauma six months prior. Her family history was non-contributory. Physical examination revealed bilateral horizontal nystagmus. Neurological examination showed left-sided dysmetria on the finger-to-nose test. Brain MRI demonstrated a signal abnormality in the left cerebellar hemisphere (Figure 1), with no significant abnormalities detected on spinal MRI. Serum anti-AQP4-IgG antibody was positive tested by V-Medical Laboratory Co., Ltd. using fixed-cell-based assays. Analysis of the cerebrospinal fluid (CSF) showed normal cell count, protein, glucose, and LDH levels. Both IgG and oligoclonal bands in blood and cerebrospinal fluid were negative. Additionally, a comprehensive autoantibody panel—testing for ANCA, anti-dsDNA, anti-Smith, anti-Scl-70, anti-Jo-1, and rheumatoid factor (RF)—was negative, with none of the markers detected. According to the 2015 International Panel for NMOSD Diagnostic Criteria for Adult Patients, the patient met all the criteria for “NMOSD with positive AQP4- IgG”, as evidenced by the presence of acute brain stem syndrome, positive AQP4-IgG, and exclusion of alternative diagnoses(1). Multiple scattered ground-glass opacities were identified on her thoracic CT, including one lesion exhibiting the “reverse halo” sign, despite her being entirely asymptomatic respiratoryly (Figure 2A). All infectious and fungal studies—including T-SPOT.TB, (1,3)-β-D-Glucan, galactomannan, and sputum culture—returned negative results. A treatment regimen of intravenous immunoglobulin and high-dose methylprednisolone, followed by rituximab, led to an improvement in her neurological symptoms. Follow-up thoracic CT at one month demonstrated significant resolution of the bilateral patchy ground-glass opacities (Figure 2B).

**Figure 3:**
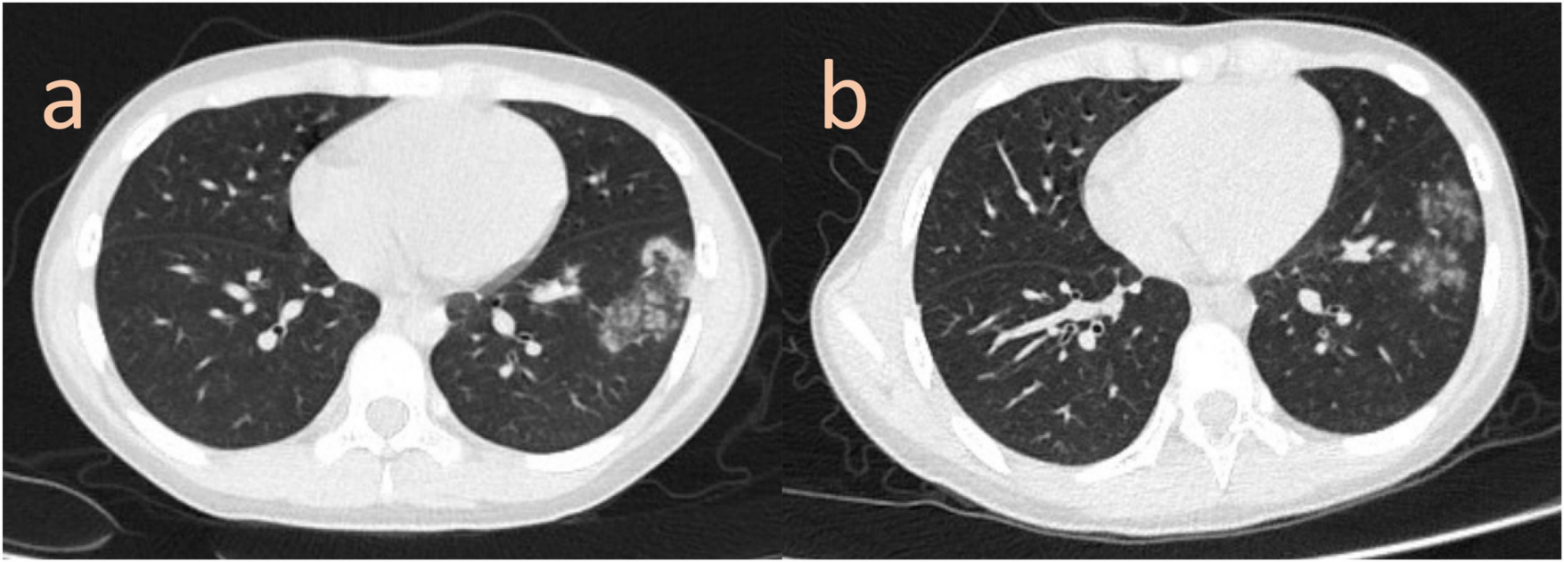
**(a)** the CT thorax demonstrated new nodules in a different location of the left lower lobe. **(b)** The lung injury resolved spontaneously after one month.

**Figure 4:**
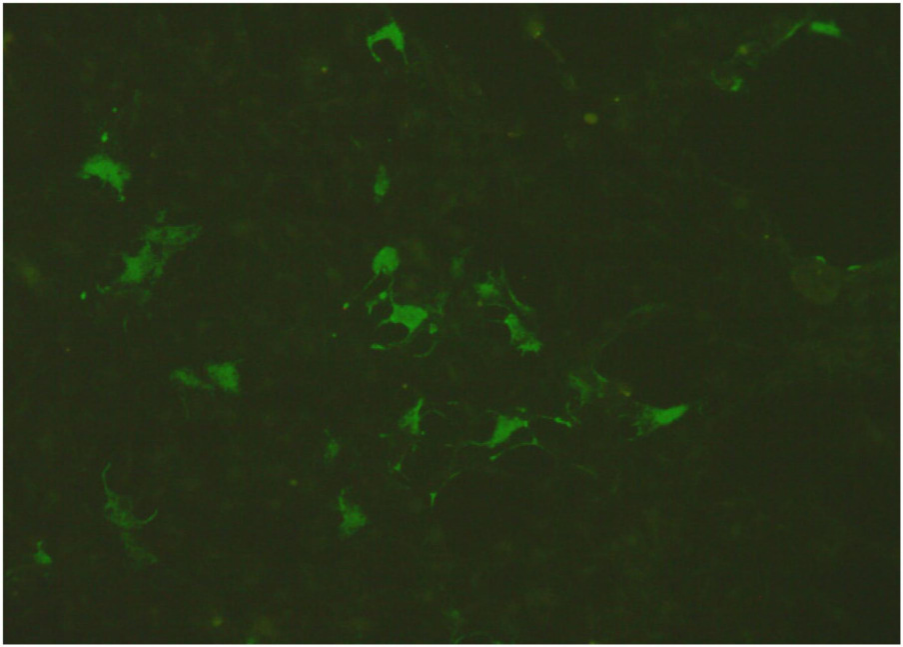
Anti-AQP4-IgG antibody in the BALF was positive for, as detected by fixed-cell-based assays at V-medical laboratory Co., Ltd.

In June 2025, while receiving the seventh scheduled dose of rituximab, the patient developed a second lung injury. She was still asymptomatic. The thoracic CT showed that new nodules had appeared in a different location of the left lower lobe (Figure 3A). Serum anti-AQP4-IgG antibody titer was 1:32. The patient underwent bronchoscopy with bronchoalveolar lavage. Microbiological examination of the bronchoalveolar lavage fluid (BALF) was negative. However, cellular analysis revealed a mixed pattern with 70% macrophages, 6% lymphocytes, and 12% neutrophils. Additionally, the BALF was positive for Anti-AQP4-IgG Antibody, and titer was 1:3.2. (Reagent kit manufacturer: Hangzhou West Friend Biotechnology Co., Ltd.; Item number: WLS-FL-1201-1005-G; The antigen-expressing cell line/clone used: HEK293.) (Figure 4). In the absence of treatment with intravenous immunoglobulin, methylprednisolone, or other immunosuppressive agents, follow-up thoracic CT one month later demonstrated spontaneous resolution of the lung injury (Figure 3B).

## Discussion

AQP4-Ab is directly pathogenic, primarily through effector mechanisms that induce astrocytopathy (9). The formation of IgG-AQP4 complexes downregulates AQP4 surface expression in central nervous system cells, triggering a cascade of events: increased blood-brain barrier permeability, complement activation, inflammatory cell recruitment, and ultimately, astrocyte damage and death (10). Although AQP4 is also widely expressed in other tissues (e.g., kidney, skeletal muscle and lung), extra-CNS manifestations of AQP4-Ab NMOSD are rare (7, 11). Reports on pulmonary involvement in NMOSD remain limited, primarily consisting of individual cases describing organizing pneumonia (12–14). This case is highly unusual, distinguishing it as the youngest documented case of early-onset NMOSD with concomitant organizing pneumonia.

Organizing pneumonia is a rare yet highly distinctive form of interstitial lung disease. This is a reversible inflammatory and fibroproliferative process, characterized by fibroinflammatory buds (Masson bodies) that do not disrupt the lung's architecture (15). Although histologic confirmation was not obtained, the clinical imaging and bronchoalveolar lavage fluid findings were highly suggestive of organizing pneumonia. The pathological relevance of complicated pulmonary disorders in anti-AQP4 antibody-positive NMOSD remains to be elucidated. Furube et al. revealed that AQP4 immunoreactivity was abundant in the structurally normal alveolar regions but severely diminished in the granulation tissue buds and epithelial cells surrounding the organizing pneumonia lesions. These same affected epithelial cells also showed positive staining for both IgG and membrane attack complex (MAC). Thus, it is plausible that the paucity of AQP4 with concomitant deposition of IgG and MAC in organizing pneumonia lesions refects the loss of AQP4 positive cells resulting from complementdependent cytotoxicity caused by the AQP4eIgG binding (16). In this case, given the patient's young age, the inherent risks of invasive procedures, and the observed improvement in lung imaging, a lung biopsy with immunohistochemical analysis was not performed. Instead, bronchoscopy with bronchoalveolar lavage was conducted, and anti-AQP4-IgG antibody testing of the BALF returned positive. There was no hemoptysis or evidence of bleeding at the time of bronchoalveolar lavage, ruling out contamination of BALF specimens by serum antibodies. This finding indirectly supports the organizing pneumonia may represent the pulmonary manifestation of AQP4-Ab NMOSD. The limitation is that multi-platform testing has not been conducted; further validation of this method requires additional cases in the future.

Cases of NMODS complicated by organizing pneumonia are relatively uncommon. They predominantly affect elderly patients, making our case particularly notable as it involves the youngest patient reported to date. This pediatric patient presented without respiratory symptoms, whereas some adults may develop cough or dyspnea (16). Similar to the 14-year-old girl reported by Shaﬁq et al. (13), the primary radiographic feature in this case was multiple scattered ground-glass opacities with a “reverse halo” sign. In adults, multifocal consolidation may also be observed in addition to these findings (8, 16). The patients showed clinical and radiological improvements following treatment with glucocorticoids and/or immunosuppressive agents (8, 13, 16). New lung lesions were observed in the later course of our case but improved spontaneously.

Owing to its rarity, there is considerable unfamiliarity with this type of lung injury among clinicians, frequently resulting in an initial misdiagnosis of infection. Additionally, pulmonary damage attributed to immunosuppressant agents, notably rituximab, must be included in the differential diagnosis. The diagnostic dilemma of whether organizing pneumonia stems from drug toxicity or NMOSD itself directly dictates the continuation or discontinuation of rituximab. To circumvent the trauma of lung biopsy, the potential of detecting Anti-AQP4-IgG Antibody in BALF as a less invasive alternative warrants further exploration.

## Conclusion

Organizing pneumonia associated with AQP4-Ab NMOSD is relatively uncommon. The case presented here represents the youngest patient reported to date. This condition can be challenging to distinguish from pulmonary infections or drug-induced lung injury caused by immunosuppressive therapy. When lung biopsy is not feasible due to clinical constraints, detection of Anti-AQP4-IgG antibody in BALF may serve as a useful diagnostic adjunct.

## Data Availability

The raw data supporting the conclusions of this article will be made available by the authors, without undue reservation.
